# Analysis, evaluation of hemodynamic differences and treatment strategy recommendations for multiple paraclinoid aneurysms

**DOI:** 10.1097/MD.0000000000050783

**Published:** 2026-09-18

**Authors:** Xianying Zhao, Jiahao Wang, Chao Gao, Shang Gao, Xuebin Wang, Xiaoliang Wang, Hailong Du, Kai Tang, Xiaolei Song, Jianliang Wu

**Affiliations:** a Graduate School, Hebei Medical University, Shijiazhuang, Hebei, China; b Department of Neurosurgery, The Second Hospital of Hebei Medical University, Shijiazhuang, Hebei, China; c Clinical Skills Training Center, The Fourth Hospital of Hebei Medical University, Shijiazhuang, Hebei, China; d Department of Foreign Languages, Hebei Medical University, Shijiazhuang, Hebei, China; e Department of Neurosurgery, HanDan Central Hospital, Handan, China.

**Keywords:** hemodynamics, intracranial aneurysm, multiple aneurysms, paraclinoid aneurysm, rupture risk

## Abstract

Multiple paraclinoid aneurysms of the internal carotid artery present distinct clinical and anatomical challenges compared with other aneurysms. This study aimed to investigate the hemodynamic characteristics of the parent artery adjacent to these lesions and to provide evidence-based suggestions for clinical treatment. We retrospectively reviewed digital subtraction angiography images of 57 patients treated between 2020 and 2024, including 18 cases with multiple paraclinoid aneurysms, 19 with a single paraclinoid aneurysm, and 20 with normal vascular anatomy. Parameters including peak systolic flow velocity and wall shear stress were derived from reconstructed hemodynamic models. We compared hemodynamic profiles among groups and evaluated the effects of simulated aneurysm obliteration on the distal vessel wall proximal to the carotid bifurcation. Four real-world follow-up cases were also analyzed for morphological and hemodynamic changes after treatment. The multiple-aneurysm group exhibited greater variability and instability in wall shear stress and flow velocity than the single-aneurysm and normal groups. Simulated aneurysm elimination showed no significant hemodynamic effect on distal vessels (*P* > .05). In clinical follow-up, however, noticeable morphological and hemodynamic alterations were observed following stent deployment. Multiple paraclinoid aneurysms induce more prominent hemodynamic disturbances in the parent artery. Aneurysm obliteration appears to have minimal immediate impact on the distal vessel wall, but stent placement may produce favorable long-term vascular remodeling, including straightened curvature and improved mild stenosis.

## 1. Introduction

Stroke remains a leading cause of morbidity and mortality worldwide. According to the Global Burden of Disease Study, the annual number of strokes and stroke-related deaths increased substantially from 1990 to 2021. In China, stroke remains one of the leading causes of death and disability-adjusted life-years, imposing an enormous disease burden on individuals, families, and society.^[1]^ Intracranial aneurysm rupture is one of the most devastating causes of hemorrhagic stroke.

Multiple paraclinoid aneurysms of the internal carotid artery are relatively rare compared with other aneurysms. The paraclinoid segment mainly refers to aneurysms occurring in the paraclinoid segment of the internal carotid artery or its adjacent areas, which are closely related to the anterior clinoid process anatomically. In a broad sense, it includes the area from the cavernous segment to the ophthalmic artery segment. The most common aneurysms in this area are ophthalmic artery aneurysms and superior hypophyseal artery aneurysms. Due to their multiple numbers, rupture can lead to more severe symptoms such as subarachnoid hemorrhage, rebleeding, vasospasm caused by hemorrhage, and delayed ischemia, and may cause compression of surrounding tissues and neurovascular structures.^[2,3]^ Its anatomical location is in the center of the skull base, covered by the anterior clinoid process and skull base dura mater, and closely related to the internal carotid artery and optic nerve, which brings great challenges to surgical clipping.^[4,5]^ In recent years, with the improvement of endovascular interventional technology, endovascular treatment has become the preferred treatment for more and more aneurysms in this area. Although endovascular treatment reduces the difficulty of treatment, it has a 4.8% adverse outcome. Considering the high risk of complications, it is crucial for us to evaluate and identify easily ruptured aneurysms before surgery and assess the balance between risks and therapeutic benefits.^[6]^ With the progress of endovascular technology in recent years, the emergence of flow diverters (FDs) has provided diversified development for the treatment of aneurysms. Compared with surgical clipping and coil embolization, simple FD implantation is beneficial to the treatment and prognosis of patients, especially for the diagnosis and treatment of multiple aneurysms, which has been recognized. Specifically, FD reduces the wall shear stress (WSS) at the aneurysm sac, diverts blood flow away from the aneurysm, and also promotes the growth of endothelial cells in the stent to repair blood vessels, which can reduce the pulsation in the aneurysm and protect the aneurysm from the severe impact of arterial blood flow.^[7]^ Hemodynamics has played an important role in the initiation, progression, and rupture of aneurysms, which has led to the development of image-based computational fluid dynamics (CFDs) in the research community. In the medical field, it enables quantitative analysis of the morphology and hemodynamics of intracranial aneurysms. It can even change the morphology of the aneurysm vessel itself to a certain extent to study the optimal treatment, eventually leading to complete occlusion and elimination of the aneurysm.^[8,9]^ The purpose of this study is to use the CFD method to analyze the changes of hemodynamic parameters around paraclinoid multiple aneurysms after their growth, and to simulate the impact on the perianeurysmal and distal blood vessels after aneurysm elimination and cure, so as to provide reference suggestions for the selection of stent coverage.

## 2. Materials and methods

### 2.1. Clinical data

We retrospectively included digital subtraction angiography (DSA) data of 57 patients with intracranial aneurysms treated in the Second Hospital of Hebei Medical University from 2020 to 2024. The inclusion criteria were: multiple and single paraclinoid aneurysms on the same vascular tree were selected and no other lesions existed around the aneurysm. The exclusion criteria were: dissecting aneurysm; pseudoaneurysm; patients with multiple intracranial stenosis; vascular stenosis rate ≥ 50% on the vascular tree where the aneurysm was located; insufficient quality of three-dimensional (3D) reconstruction for hemodynamic analysis; and lack of original data.

### 2.2. Construction of 3-dimensional geometric model

#### 2.2.1. Construction of geometric model

The original DSA data (DICOM format) were imported into 3D Slicer 5.2.2, and preliminary modeling was performed using threshold segmentation and other modes.

The model was processed in 3-matic Medical 13.0 (x64) to obtain a relatively smooth 3D model. To ensure the similarity of the inflow and outflow ports and avoid errors, the proximal end was cut to approximately the starting point of the cavernous segment of the internal carotid artery, and the distal end was cut to the bifurcation of the internal carotid artery. At the same time, post-processing was performed on the multiple aneurysm model to erase the aneurysms, achieving an effect similar to occlusion and cure.

### 2.3. Finite element model

#### 2.3.1. Mesh generation

The model was imported into ICEM CFD 19.2 (ANSYS, Inc.) software to set boundary layer meshes, and volume mesh and boundary layer mesh models were generated.

#### 2.3.2. Material properties and boundary conditions

The meshed model was imported into CFX-pre 19.2 for parameter setting. Blood was assumed to be an adiabatic, uniform, isotropic, and incompressible Newtonian fluid, and its flow mode was pulsatile laminar flow. The density of blood was set to 1050 kg/m^3^, and the kinematic viscosity coefficient was 0.0035 Pa·s. The model was set as steady flow. The boundary condition at the inlet referred to the maximum systolic peak velocity of 0.41 m/s in the velocity curve of 1 cardiac cycle in the literature. The outlet adopted a free outflow boundary condition, and the working pressure was set to the average intra-arterial pressure of 13,332.24 Pa (100 mm Hg). The influence of gravity was not considered, and the vascular wall was set as a nonslip rigid wall. The maximum number of iterations per time step was 200, and the average output number of iterations was 25.^[10]^ After output, post-processing was performed in CFX-post, and finally, values such as maximum systolic peak WSS and blood flow velocity, as well as the corresponding cloud maps and curves, were output.

## 3. Statistical analysis

Output variables were collected, sorted, and imported into IBM SPSS (IBM Corp.) Statistics 21 for data analysis. Box plots were used for the comparison of multiple groups of data to compare the average level and variation degree, presenting the mean value, interquartile range, maximum value, and minimum value to show the degree of data dispersion. When comparing the preprocessing and post-virtual results, the Mann–Whitney *U* test (for non-normally distributed data) and error bar charts were used for mean comparison, and inferential analysis was performed on the test of 2 independent samples, with *P* < .05 considered statistically significant.

## 4. Results

### 4.1. Collection results and epidemiological characteristics

Clinical data of patients were selected for statistics. A total of 3 groups of models were included to obtain original 3D data of 57 patients, including 37 patients with aneurysms (7 patients with bilateral aneurysms, 11 males and 18 females), 18 models of multiple aneurysms (all unruptured), 19 cases of single aneurysm (all unruptured), and past medical history, etc, as shown in Table 1.

**Table 1:** Comparison of clinical information between multiple aneurysm cases and single aneurysm cases.

Number	Gender	Age	Past history	Number of aneurysms	Location type	Rupture state
1	Female	64	Hypertension	2	Bilateral	Unruptured
2	Female	72	Hypertension	2	Bilateral	Unruptured
3	Male	46	Hypertension	3	Unilateral	Unruptured
4	Female	73	Hypertension	2	Unilateral	Unruptured
5	Male	51	Hypertension	2	Bilateral	Unruptured
6	Male	66	Hypertension, diabetes	2	Bilateral	Unruptured
7	Female	65	Hypertension	3	Unilateral	Unruptured
8	Female	68	Hypertension, coronary heart disease	2	Unilateral	Unruptured
9	Female	65	None	2	Unilateral	Unruptured
10	Female	50	Hypertension	2	Unilateral	Unruptured
11	Female	63	Hypertension	2	Unilateral	Unruptured
12	Female	47	None	3	Unilateral	Unruptured
13	Female	60	Hypertension	2	Unilateral	Unruptured
14	Female	59	Hypertension	1	Unilateral	Unruptured
15	Female	74	Hypertension	1	Unilateral	Unruptured
16	Male	45	Hypertension	1	Bilateral	Unruptured
17	Female	49	None	1	Bilateral	Unruptured
18	Male	49	None	1	Unilateral	Unruptured
19	Female	62	None	1	Unilateral	Unruptured
20	Male	57	None	1	Unilateral	Unruptured
21	Male	74	None	1	Unilateral	Unruptured
22	Male	54	None	1	Unilateral	Unruptured
23	Male	58	None	1	Unilateral	Unruptured
24	Male	68	Hypertension, diabetes	1	Bilateral	Unruptured
25	Female	39	Hypertension	1	Unilateral	Unruptured
26	Female	68	Hypertension	1	Unilateral	Unruptured
27	Female	55	Hypertension	1	Unilateral	Unruptured
28	Male	70	Hypertension	1	Unilateral	Unruptured
29	Female	49	Hypertension	1	Unilateral	Unruptured

### 4.2. Comparison of hemodynamic results among groups

**Figure 1.**
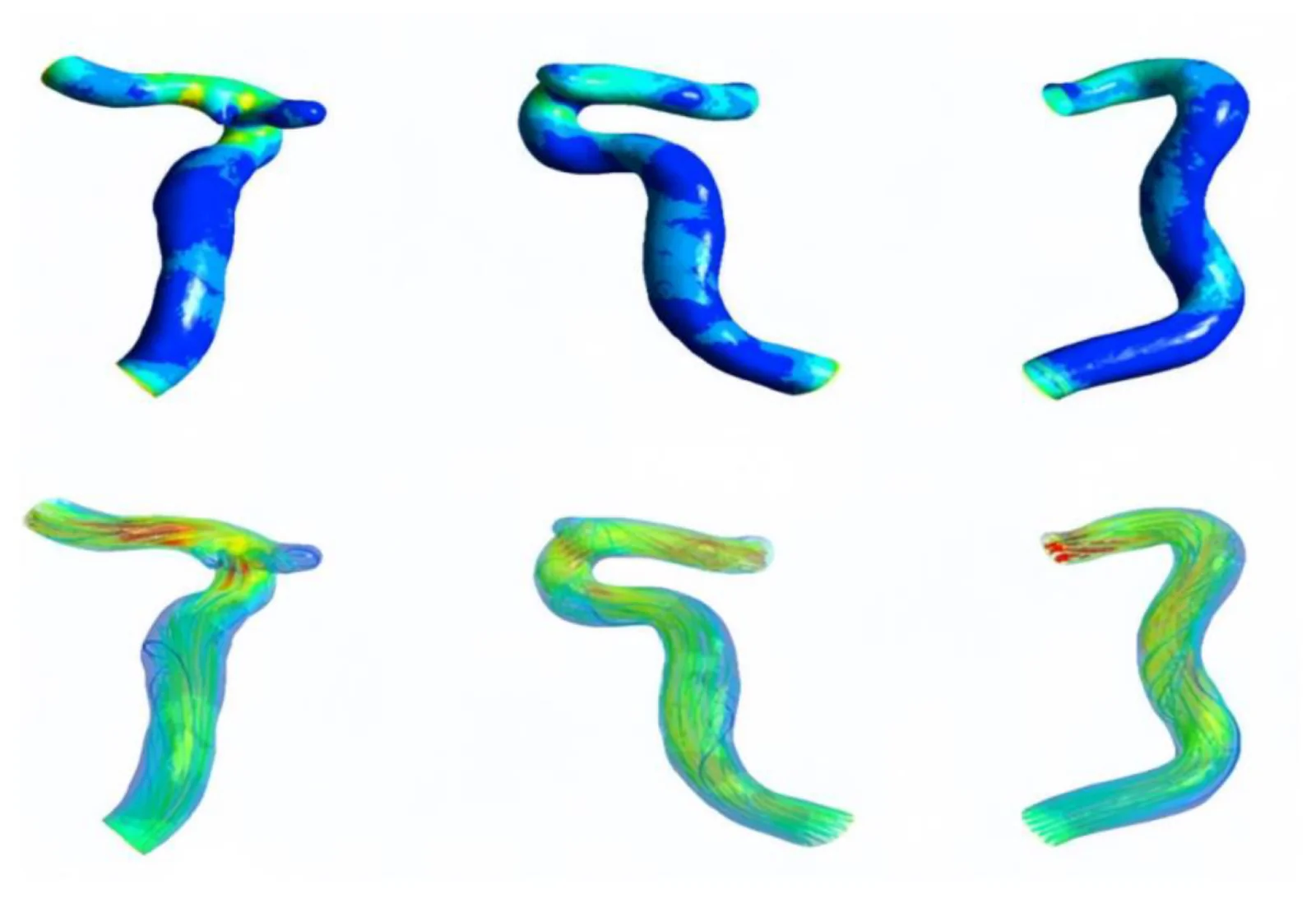
Comparison of wall shear stress (WSS) and blood flow velocity among the 3 groups of models. WSS = wall shear stress.

**Figure 2.**
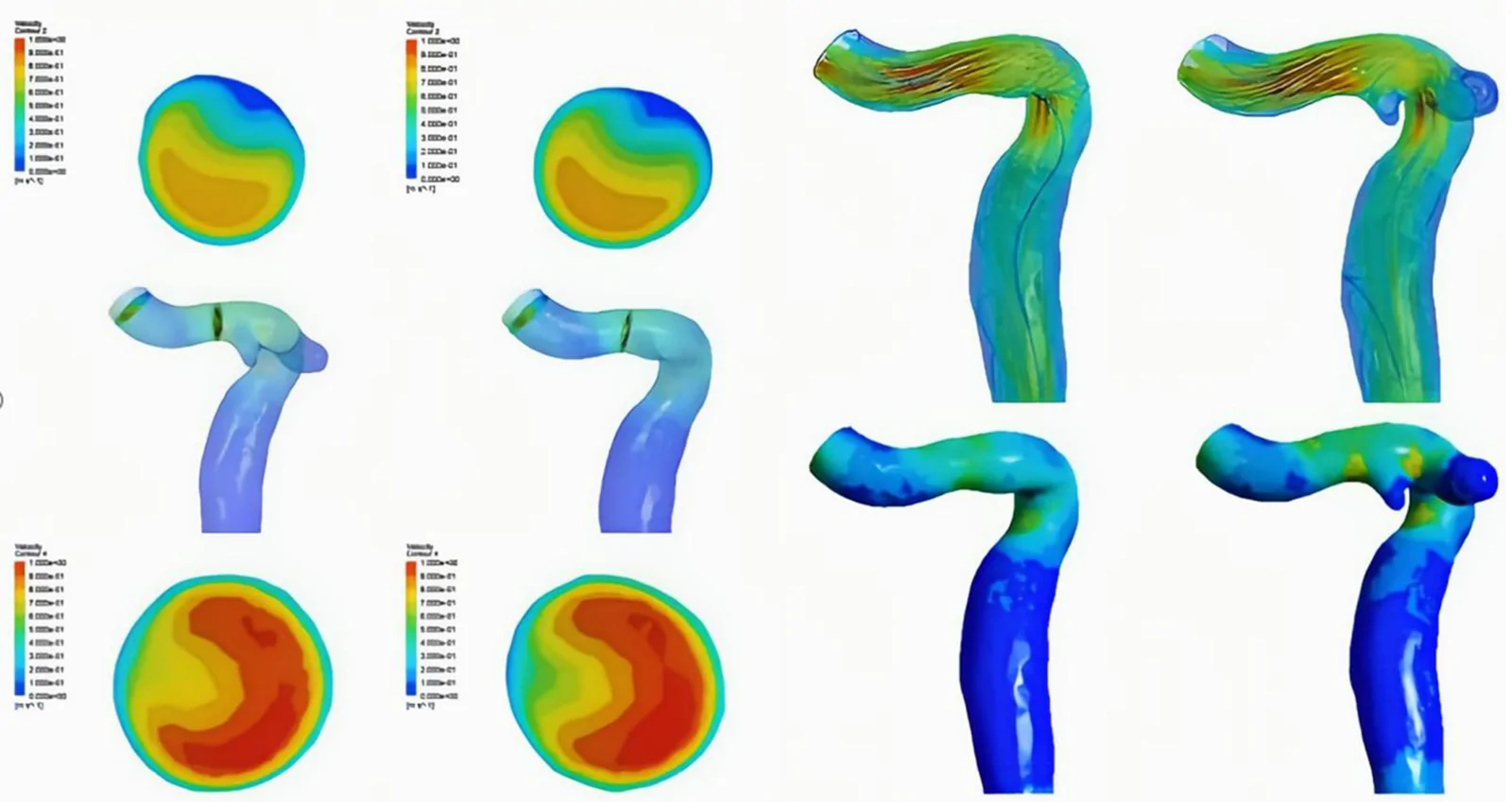
Impact of the 3 groups of models on wall shear stress (WSS). WSS = wall shear stress.

The parameters WSS and blood flow velocity of the 3 groups of blood vessels from the start of the cavernous segment to the end of the internal carotid artery were compared, and the output mode diagram is shown in Figure 1. It shows that with the increase in the number of aneurysms, the surrounding WSS, blood flow velocity, and other hemodynamic parameters have significant differences, and the degree of difference interval is positively correlated, as shown in Figure 2. The WSS change at the neck of multiple aneurysms is greater, and the blood flow velocity is faster.

### 4.3. Comparison between simulated multiple aneurysm elimination and original vascular model

**Figure 3.**
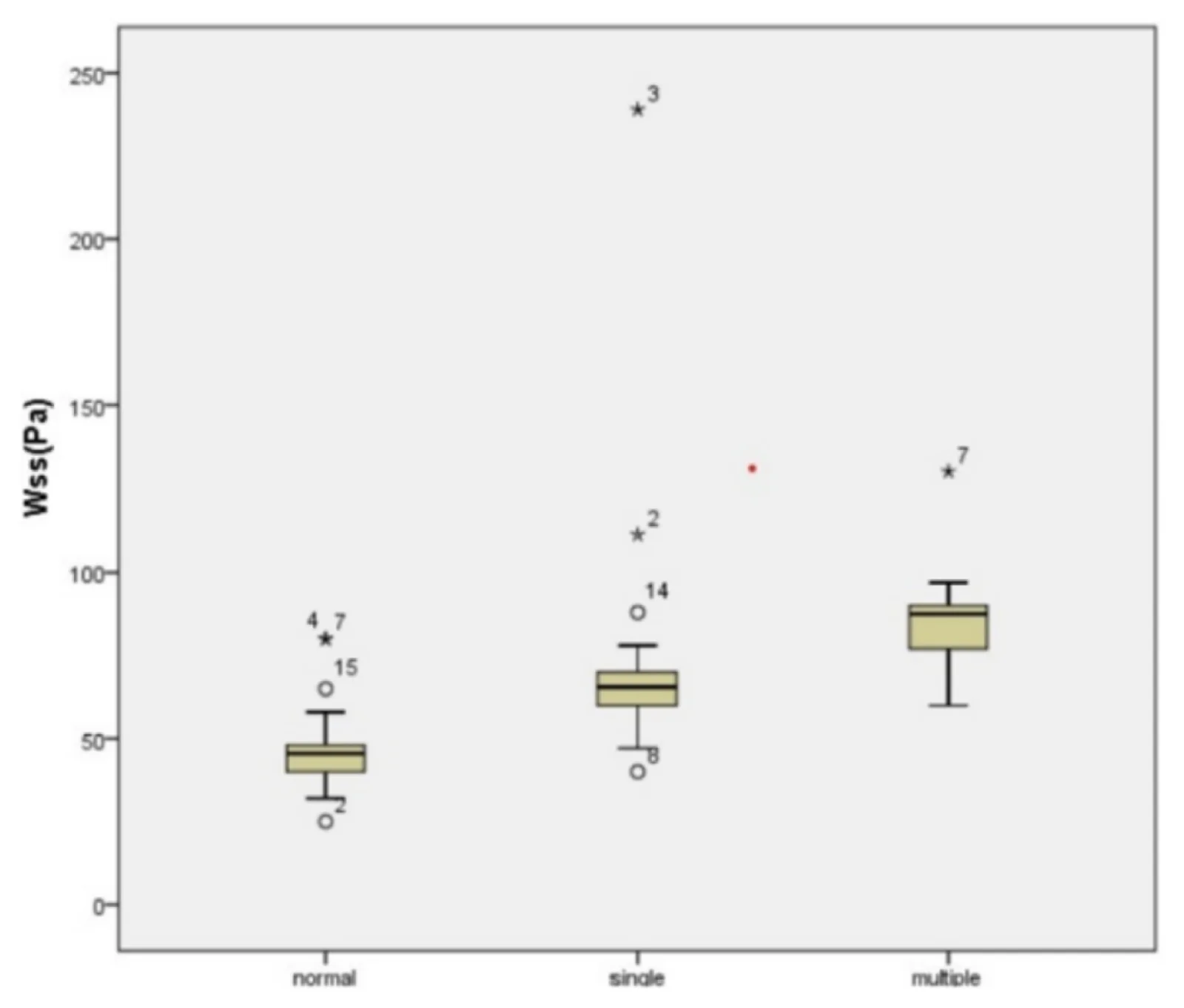
Comparison of wall shear stress (WSS) and blood flow velocity between the simulated aneurysm elimination group and the original group. WSS = wall shear stress.

We selected 10 models of multiple aneurysms (excluding models with excessive differences in the clinoid bend and posterior communicating segment) and performed simulated aneurysm elimination steps, with the output mode diagram shown in Figure 3.

**Figure 4.**
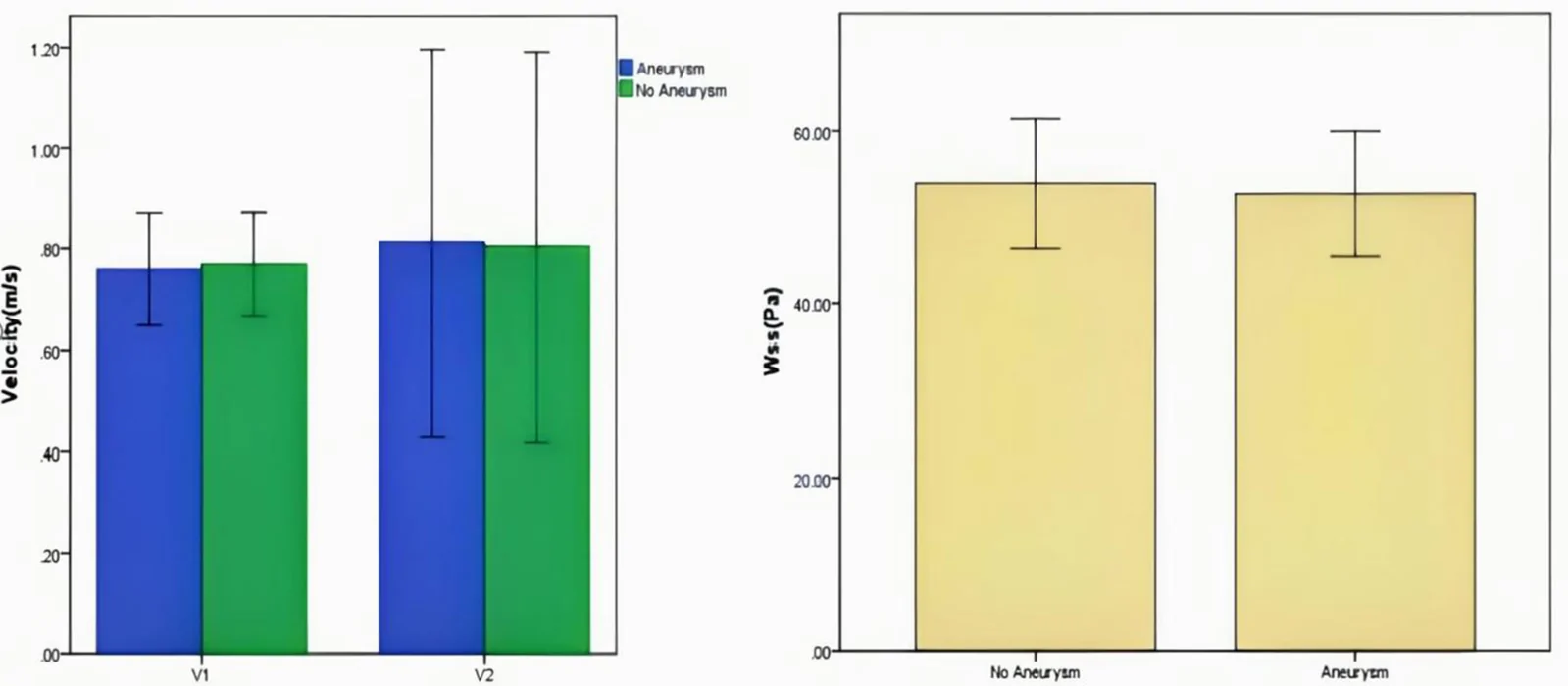
Comparison of wall shear stress (WSS) and blood flow velocity data between the simulated group and the original group. WSS = wall shear stress.

Comparison of WSS and blood flow velocity showed no significant difference in the impact on the distal vascular wall and no obvious increase in data, as shown in Figure 4. The blood flow inlet and outlet distal to the most distal aneurysm were intercepted, and the maximum systolic peak WSS and blood flow velocity of the 2 groups of data were subjected to statistical analysis, showing *P* > .05, indicating no significant statistical difference.

### 4.4. Comparison between real-world data and simulated data

**Figure 5.**
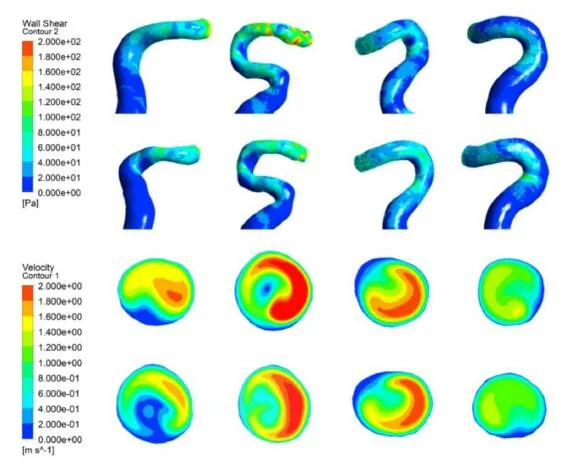
Comparison between real-world follow-up data and simulated data.

We selected DSA data of 4 followed-up cases included in our study (half a year after surgery). The results showed that with stent implantation, the reexamined models had morphological changes (stretching of vascular curvature and repair of mild stenosis), and visually observable changes also appeared in distal blood flow velocity and WSS, as shown in Figure 5.

## 5. Discussion

Literature reports on multiple aneurysms are relatively rare. In this study, we focused mainly on multiple paraclinoid aneurysms. Due to their deep location, such aneurysms are more suitable for endovascular interventional treatment compared with craniotomy, so the analysis of hemodynamic parameters is more important. We selected 3 groups (normal, single, and multiple aneurysms) for comparison of the same vascular segment and obtained corresponding data. This showed that compared with the other 2 groups of models, multiple aneurysms had a greater impact on the hemodynamic parameters of the blood vessels around the aneurysm, and the numerical changes around them were more drastic. Aneurysm rupture is the most common cause of nontraumatic subarachnoid hemorrhage, and hemodynamics is considered one of the many factors leading to aneurysm rupture. Image-based CFD models have identified the connection between the hemodynamics of intracranial aneurysms and their growth and rupture potential.^[11,12]^ According to relevant literature, although hemodynamic-related issues indicate the rupture risk of aneurysms, factors such as morphology and past medical history cannot be ignored.^[13]^ Research on the occurrence and development of aneurysms is constantly updated, and both morphology and hemodynamics are related to rupture during the process.^[14,15]^ However, the long-term evolution of aneurysms remains unpredictable, and our cross-sectional findings may not fully reflect future hemodynamic changes. At the same time, our center followed up and compared the reexamination data of 4 real-world cases, and it can be seen that with stent implantation, the morphology of the blood vessels has changed. We simply simulated the WSS of the clinoid segment and the distal blood flow velocity according to the same methods and parameters, and visually observable differences were also found. However, due to the insufficient sample size, only partial descriptions were made, so no further statistical verification was performed. At the same time, problems such as operational differences and vasospasm brought difficulties to our study. Histological progress studies have found that during the growth of aneurysms, the formation and growth of aneurysms mainly occur in the high WSS area of the vascular wall. The growth of the aneurysm wall requires smooth muscle cell proliferation, which continuously repairs and maintains sufficient strength to adapt to hemodynamic pressure.^[16]^ Previous studies have reported that both low WSS and high WSS of ruptured aneurysms are related to the rupture state. However, the final research results all agree that complex blood flow structures occur more often in ruptured aneurysms.^[17,18]^ Therefore, we can speculate that multiple aneurysms, due to the repeated occurrence of high and low WSS around them, will lead to more serious outcomes.

Regarding its treatment, we roughly believe that the essence of treatment is to eliminate the impact of hemodynamic parameters such as WSS on blood vessels. Placing stents in areas with less impact will reduce the WSS change of the entire segment near the aneurysm and reduce the probability of late recurrence. Therefore, we simulated the model after the elimination of multiple aneurysms and compared it with the original model. However, the conclusion we drew was that after becoming a nearly smooth blood vessel, the WSS near the clinoid bend changed correspondingly, while the changes in distal WSS and blood flow velocity were not obvious. After intercepting the maximum systolic peak WSS and blood flow velocity of the distal blood vessel and performing a rank sum test, it showed *P* > .05, which was not statistically significant. The results showed that the elimination of the aneurysm had no obvious impact on the hemodynamics under this model (before the bifurcation of the internal carotid artery). The local difference may also be related to the simulated elimination of the aneurysm neck. Thenier-Villa et al showed in a study that after treatment of the proximal aneurysm, the hemodynamic changes of the distal aneurysm are related to the theoretically increased risk of bleeding.^[19]^ This study verified that there is an impact on the aneurysm at the distal middle artery after treatment of the proximal aneurysm. We can suspect that in this case, the change of the proximal end has an impact on the hemodynamics of the distal complex pathway, but compared with the case where there is a lesion in the distal end, the changes in WSS and blood flow velocity of the normal vascular wall are not significant. So far, we can temporarily draw this conclusion.

The rise of endovascular treatment has brought new directions for the treatment of such diseases, followed by discussions on posttreatment complications. Regarding the impact on perforating vessels, previous studies have shown that after oral administration of standard dual antiplatelet drugs, when treating aneurysms that require releasing an FD to cover the opening of the anterior cerebral artery, preoperative patency of the anterior communicating artery has a significant impact on the prognosis of the anterior cerebral artery after surgery, and it becomes more obvious with the extension of follow-up time. These patients with preoperative patency of the anterior communicating artery are more likely to experience reduction or even occlusion, and oversized devices and shorter stents can reduce the risk of ischemic injury.^[20,21]^ At the same time, another study on the blood flow velocity of the ophthalmic artery before and after stent release showed that hemodynamic changes such as decreased blood flow are not the cause of ophthalmic artery occlusion and other complications after stent release, and the formation of neointima may be the cause of such occlusion. It can be seen that with the emergence of FD in particular,^[22]^ we can make decisions according to specific intraoperative conditions when choosing treatment, including the protection of branch vessels and the impact on the middle cerebral artery and anterior cerebral artery, to avoid the impact of complications such as delayed cerebral ischemia.^[23,24]^ However, Berg and Beuing stated in their study that we pay too much attention to the research of a certain limited area and ignore the impact of the entire segment of blood vessels, which is also the limitation encountered in our study. The selected area focuses on the vascular problems of a certain area,^[25]^ and the evolution, location, and angle of the aneurysm all need further research.

## 6. Conclusion

As a special type of aneurysm, multiple paraclinoid aneurysms have a more obvious impact on hemodynamic changes of blood vessels due to their location and the increase in the number of aneurysms. The reduction or disappearance of aneurysms may have no obvious impact on the distal vascular wall (before the bifurcation of the internal carotid artery), but with the intervention of stent release, there may be long-term effects on the blood vessels (stretching of vascular curvature and repair of mild stenosis).

## Acknowledgments

The authors would like to express their sincere gratitude to all individuals who contributed to the completion of this study.

## Author contributions

**Data curation:** Xiaoliang Wang, Hailong Du, Xiaolei Song.

**Methodology:** Kai Tang.

**Software:** Chao Gao.

**Supervision:** Shang Gao.

**Visualization:** Xuebin Wang.

**Writing – original draft:** Xianying Zhao, Jianliang Wu.

**Writing – review & editing:** Jiahao Wang, Jianliang Wu.
